# Limited polymorphism in *Plasmodium falciparum *ookinete surface antigen, von Willebrand factor A domain-related protein from clinical isolates

**DOI:** 10.1186/1475-2875-5-55

**Published:** 2006-07-05

**Authors:** Jack S Richards, Nicholas J MacDonald, Damon P Eisen

**Affiliations:** 1 Victorian Infectious Diseases Service, Royal Melbourne Hospital, Parkville, Victoria, Grattan St, Parkville, Victoria, 3050, Australia; 2 Malaria Vaccine Development Branch, NIAID, National Institutes of Health, Rockville, Maryland, USA

## Abstract

**Background:**

As malaria becomes increasingly drug resistant and more costly to treat, there is increasing urgency to develop effective vaccines. In comparison to other stages of the malaria lifecycle, sexual stage antigens are under less immune selection pressure and hence are likely to have limited antigenic diversity.

**Methods:**

Clinical isolates from a wide range of geographical regions were collected. Direct sequencing of PCR products was then used to determine the extent of polymorphisms for the novel *Plasmodium falciparum *sexual stage antigen von Willebrand Factor A domain-related Protein (PfWARP). These isolates were also used to confirm the extent of diversity of sexual stage antigen Pfs28.

**Results:**

PfWARP was shown to have non-synonymous substitutions at 3 positions and Pfs28 was confirmed to have a single non-synonymous substitution as previously described.

**Conclusion:**

This study demonstrates the limited antigenic diversity of two prospective *P. falciparum *sexual stage antigens, PfWARP and Pfs28. This provides further encouragement for the proceeding with vaccine trials based on these antigens.

## Background

Development of effective *Plasmodium falciparum *vaccines is a global health priority with all stages of the malaria lifecycle being investigated for possible vaccine strategies [1]. Mosquito-stage vaccines aim to induce immunity to the forms of the parasite found in the mid-gut of mosquitoes: the sexual stage macro- and microgametes and the post-fertilization zygotes and ookinetes. Antibodies that target antigens in these stages prevent infection in the mosquito thus breaking the transmission cycle. Since malaria transmission is both highly localized and focal [2], such vaccines may form a useful element as part of integrated control programmes to locally eliminate or substantially reduce transmission. They may also have a useful role in conjunction with chemotherapeutic agents to prevent drug-resistant or vaccine-escape mutants.

The *P. falciparum*, mosquito-stage vaccine candidate antigens include Pfs25, Pfs28, Pfs48/45, Pfs230 and the recently described, von Willebrand factor A domain-related protein (PfWARP) [3,4]. PfWARP (PF08_0136b) is located on chromosome 8 (GenBank: NC_004329 Region: 1221207...1222079) and encodes a 290 amino acid protein. On the basis of previous study of orthologues in *Plasmodium berghei *and *Plasmodium gallinaceum*, PfWARP is thought to be a soluble micronemal protein expressed in the late ookinete and early oocyst [5-7]. It is proposed that the von Willebrand factor A domains found in PfWARP and other malaria antigens, including circumsporozoite protein and thrombospondin-related anonymous protein have a role as an adhesive substrate and may assist in host cell invasion [7,8].

Pfs28 (PF10_0302) is a lead candidate for a transmission-blocking vaccine. It is a 28-kDa protein and is presumably anchored to parasite surface by glycosylphosphatidylinositol [9]. It is encoded on chromosome 10 and shares putative functional determinants with Pfs25. Pfs28 is expressed on the surface of late ookinetes of *P. falciparum *and it is proposed that it is involved in adherence to the mosquito's gut epithelium [10]. Previous studies have examined Pfs28 polymorphism in laboratory isolates Dd2, 2D11, CAMP, LE5, LF4, HB3, 7G8 [11] and from 32 field isolates from a geographically restricted region in the Philippines [12]. These have shown the presence of a single non-synonymous A274G base substitution, resulting in a conservative amino acid change from lysine to arginine (GenBank: L25843).

As part of the assessment of PfWARP's suitability as a vaccine candidate, the diversity in this gene was assessed using *P. falciparum *field isolates from a range of geographic locations. In addition, Pfs28 was sequenced in the same isolates to extend previous observations of its' diversity.

## Methods

Review and approval of the study was obtained from the Research Ethics Committee of the Royal Brisbane Hospital and the Melbourne Health Human Research Ethics Committee. Clinical samples from *P. falciparum*-infected patients were obtained from the Malaria Reference Laboratory at the Royal Brisbane Hospital, Queensland. Twenty-two samples were studied from unrelated travellers returning from Papua New Guinea, Solomon Islands, Pakistan, Kenya, Tanzania, Uganda, Zimbabwe, and Ghana.

Whole blood specimens were stored in 8 M guanidine hydrochloride/0.1 M Na H_2_PO_4_. Genomic DNA was isolated from the samples using the QIAquick^® ^PCR purification kit (Qiagen, Hilden, Germany). PfWARP and Pfs28 polymorphism was assessed using PCR conditions as outlined (Table 1). All PCR reactions used Amplitaq Gold^® ^DNA polymerase (Roche Diagnostics, Mannheim, Germany). PCR products were detected after submarine electrophoresis using 1% agarose gel stained with ethidium bromide. PCR products were purified for direct sequencing using QIAquick^® ^PCR purification kit (Qiagen, Hilden, Germany) or were agarose gel purified using MinElute Gel extraction kit^® ^(Qiagen, Hilden, Germany) purification kit. DNA sequencing was performed utilising the automated 3100 Genetic Analyser capillary system. In addition, samples with multiple infections of *P. falciparum *were detected by merozoite surface protein 2 (MSP2) PCR as previously described [13]. Amplification of this highly polymorphic gene allowed rapid detection of size separable fragments by gel electrophoresis.

**Table 1:** Details of primers, primer and MgCl_2 _concentrations and cycle conditions for the amplification of PfWARP and Pfs28 genes. Oligonucleotide positions are based on NCBI Genebank sequence PfWARP (NC_004329) and Pfs28 (L25843).

Oligonucleotide	Nucleotide sequence	Description	Primer (μM)/MgCl_2 _(mM)	Cycle conditions
PfWARP_USF	GTTGTTGTATAATAAGAGAGAGAAAAATG	WARP bases 1221181 to 1221209	0.6/2.0	94°C 10 min (94°C 30 sec, 52°C 30 sec, 72°C 60 sec) 40 cycles, 72°C 5 min
PfWARP_IR	TACATCTTATGATTTATTCTTATCACATA	WARP bases 1222057 to 1222085		
PfWARP_IF	GTGGTATTATGTTTGGGTATGATATCAGC	WARP bases 1221222 to 1221250	0.6/1.5	94°C 10 min (94°C 30 sec, 52°C 30 sec, 72°C 60 sec) 25 cycles, 72°C 5 min
PfWARP_IR	TACATCTTATGATTTATTCTTATCACATA	WARP bases 1222057 to 1222085		
Pfs28_F1	ATGAATACATATTTTAAGGTACTTCTT	Pfs28 bases 60 to 86	1.0/2.5	94°C 10 min (94°C 60 sec, 46°C 60 sec, 72°C 45 sec) 40 cycles, 72°C 10 min
Pfs28_R650	GAGCATACAATCAGAACGTGTGTTAGG	Pfs28 bases 680 to 709		
Pfs28_F40	CAACTTTACATAACGTTGAATAAGGCTC	Pfs28 bases 99 to 126	1.0/2.5	94°C 10 min (94°C 60 sec, 50°C 60 sec, 72°C 45 sec) 34 cycles, 72°C 10 min
Pfs28_R630	GCATACAATCAGAACGTGTGTTAG	Pfs28 bases 666 to 689		

## Results

PfWARP was amplified from 19 of the field samples and three laboratory strains (3D7, FC27, and FCR3). As judged by MSP2 genotyping, there were multiple infections in 7 of the 19 patient samples. These 7 patient samples had a minimum of 26 genetically different clones of *P. falciparum*. However, direct sequencing of PCR products from each of these samples showed only a single genotype of PfWARP with no mixed peaks in the sequencing traces. Non-synonymous nucleotide substitutions were found to occur in three positions amongst the variant sequences. No synonymous substitutions were identified. The predicted amino acid changes were conservative and contained within the single Von Willebrand factor domain (Table 2). Sample numbers were insufficient to determine whether there was any geographic distribution to the PfWARP alleles.

**Table 2:** Details of PfWARP and Pfs28 polymorphisms and geographic origin for each isolate

Isolate number	Geographic origin^a^	PfsWARP sequence^b^			Pfs28 sequence^c^
		
		Nucleotide sequence 1221576–1221581	Nucleotide sequence 1221645–1221650	Nucleotide sequence 1221885–1221890	Nucleotide sequence 270–275
3D7	Netherlands	GTC**T**TT	GTA**G**T**C**	CAA**A**CA	TTTA**A**A
FC27	Papua New Guinea	GTC**C**TT	GTA**T**T**C**	CAA**G**CA	X^d^
FCR3	Central/South America	GTC**C**TT	GTA**T**T**C**	CAA**G**CA	X^d^
1	Uganda	GTC**T**TT	GTA**C**T**C**	CAA**A**CA	TTTA**A**A
2	Papua New Guinea	GTC**C**TT	GTA**T**T**C**	CAA**G**CA	TTTA**G**A
3	Papua New Guinea	GTC**C**TT	GTA**T**T**C**	CAA**G**CA	TTTA**G**A
4	Ivory Coast	GTC**T**TT	GTA**T**T**G**	CAA**A**CA	X^d^
5	Papua New Guinea	GTC**C**TT	GTA**T**T**C**	CAA**G**CA	TTTA**G**A
6	Papua New Guinea	GTC**C**TT	GTA**T**T**C**	CAA**G**CA	X^d^
7	Tanzania	GTC**C**TT	GTA**T**T**C**	CAA**G**CA	TTTA**G**A
8	Tanzania	GTC**C**TT	GTA**T**T**C**	CAA**G**CA	TTTA**A**A
9	Papua New Guinea	GTC**C**TT	GTA**T**T**C**	CAA**G**CA	X^d^
10	Solomon Islands	GTC**C**TT	GTA**T**T**C**	CAA**G**CA	TTTA**G**A
11	Ghana	GTC**T**TT	GTA**T**T**G**	CAA**A**CA	TTTA**A**A
12	Papua New Guinea	GTC**C**TT	GTA**T**T**C**	CAA**G**CA	TTTA**G**A
13	Papua New Guinea	GTC**T**TT	GTA**G**T**C**	CAA**A**CA	TTTA**G**A
14	Papua New Guinea	GTC**T**TT	GTA**T**T**G**	CAA**G**CA	TTTA**A**A
15	Pakistan	GTC**C**TT	GTA**T**T**C**	CAA**G**CA	TTTA**G**A
16	Papua New Guinea	GTC**C**TT	GTA**T**T**C**	CAA**G**CA	TTTA**G**A
17	Papua New Guinea	GTC**C**TT	GTA**T**T**C**	CAA**G**CA	TTTA**G**A
18	Kenya	GTC**T**TT	GTA**G**T**C**	CAA**A**CA	TTTA**A**A
19	Papua New Guinea	GTC**C**TT	GTA**T**T**C**	CAA**G**CA	TTTA**A**A
20	Papua New Guinea	X^d^	X^d^	X^d^	TTTA**G**A
21	Togo	X^d^	X^d^	X^d^	TTTA**A**A
22	Papua New Guinea	X^d^	X^d^	X^d^	TTTA**A**A
23	Tanzania	X^d^	X^d^	X^d^	TTTA**A**A
24	Zimbabwe	X^d^	X^d^	X^d^	TTTA**A**A
25	Ghana	X^d^	X^d^	X^d^	TTTA**A**A
CAMP^e^	SE Asia	X^d^	X^d^	X^d^	TTTA**A**A
LE5^e^	Africa	X^d^	X^d^	X^d^	TTTA**A**A
LF4^e^	Africa	X^d^	X^d^	X^d^	TTTA**A**A
HB3^e^	Central/South America	X^d^	X^d^	X^d^	TTTA**A**A
7G8^e^	Central/South America	X^d^	X^d^	X^d^	TTTA**A**A
Dd2^e^	SE Asia	X^d^	X^d^	X^d^	TTTA**G**A
2D11^e^	SE Asia	X^d^	X^d^	X^d^	TTTA**G**A
2 Philippine isolates^e^	SE Asia	X^d^	X^d^	X^d^	TTTA**A**A
30 Philippine isolates^e^	SE Asia	X^d^	X^d^	X^d^	TTTA**G**A

^a ^The precise geographic origin of laboratory parasite strains is unclear in some instances

^b ^Variable nucleotide positions for PfWARP are indicated in bold (GenBank: NC_004329 Region: 1221207...1222079).

^c ^Variable nucleotide positions for Pfs28 are indicated in bold (GenBank: L25843).

^d ^The symbol "X" is used to indicate that the isolate was not tested.

^e ^Theses isolates, laboratory strains and their geographic origin are cited as given in the paper by Hafalla et al [12].

Pfs28 was amplified in 22 field samples (representing at least 31 clones due to the existence of multiple MSP2 alleles in nine patients) and in 3D7. The A274G, non-synonymous, nucleotide substitution as previously described, was found with 11 of the 22 samples having the A274 allele (Table 2). No other nucleotide substitutions were present. This data was combined with that from Hafalla et al [12] to consider the geographic distribution of Pfs28 alleles. In Asia/Pacific isolates, 42 of 48 carried the G274 allele while only 1 of 14 of Africa/Americas isolates bore this allele (p < 0.001, Fisher's exact test). While recognizing the size limitations of this study, it is still striking that there is such a significant difference in the geographic distribution of these Pfs28 alleles (Table 2).

## Discussion

For the first time, this study addresses the issue of antigenic diversity in PfWARP. This antigen appears to have restricted diversity with infrequent point mutations as defined. Additionally, by documenting the genotype of Pfs28 in field isolates from a larger cohort with wider geographic distribution than in previous studies, we confirm that there are only two alleles of this protein. Restricted antigenic diversity has been described in other sexual stage antigens and is explicable due to the absence of human immune selection pressure [14-16]. By contrast, asexual stage candidate antigens such as MSP2 are highly polymorphic due to *P. falciparum*'s immune evasion mechanisms [17].

Pfs28, and its orthologues from other species, Pvs28 (*Plasmodium vivax*) and Pbs21 (*P. berghei*) clearly induce antibodies that block parasitic infection in mosquitoes and are thus candidate antigens for inducing transmission blocking immunity in humans either alone [18] or in combination with other mosquito-stage antigens. So far there have been two published vaccine trials in humans with Pfs25 [19] and with its *P. vivax *orthologue, Pvs25 [20], with other trials currently underway.

## Conclusion

The demonstration of limited sequence variation in the newly described *P. falciparum *ookinete surface antigen PfWARP and the confirmed highly conserved nature of Pfs28 in clinical samples from a wide geographical distribution, increase the confidence that these proteins may also be suited for mosquito-stage vaccine development.

## Authors' contributions

JSR carried out the genetic studies, sequence alignment and drafted the manuscript. NJM designed and supplied the primers. DPE designed a study of returned travellers with malaria and recruited the patients, assisted in the sequence alignment and helped draft the manuscript. All authors read and approved the final manuscript.
